# Alternating temperatures affect the performance of *Trichogramma* species

**DOI:** 10.1093/jis/14.1.41

**Published:** 2014-01-01

**Authors:** D. M. Firake, M. A. Khan

**Affiliations:** 1 Division of Entomology, ICAR Research Complex for NEH Region, Umiam, Meghalaya-793103, India; 2 Department of Entomology, G.B. Pant University of Agriculture and Technology, Pantnagar, Uttarakhand, India

**Keywords:** biological attributes, egg parasitoids, temperature shocks, *Trichogramma chilonis*, *Trichogramma poliae*

## Abstract

The environmental compatibility of a biological control agent is an important aspect of successful reduction of agricultural pests. Temperature fluctuations during the day have a strong influence on the performance of laboratory-reared parasitoids. In field conditions, *Trichogramma* (Hymenoptera: Trichogrammatidae) wasps are exposed to variable temperatures during their development, which has a significant impact on their ability to control pest species. A simulation-based study was undertaken to evaluate the impact of variations in daily temperature on the pest-control abilities of female *Trichogramma* and their immature progenies. Considering the temperature variability of different agricultural zones of India, five temperature levels ranging from 20ºC to 40ºC were selected for daily short-term heat shocks to the immature progenies and egg-laying females of two major *Trichogramma* species. Intensity and frequency of thermal shocks showed inverse relationships with adult emergence, fecundity, and longevity of *T. chilonis* and *T. poliae* . Parasitoid pupae were found to be more tolerant to temperature variations than eggs and larvae. Fecundity and longevity of parasitoids were significantly reduced under high temperature shocks to egg-laying females. Sex ratio was significantly affected by high temperature shocks to the immature and adult stages. However, the effect was more severe in eggs. A female-biased sex ratio was apparent in both parasitoids throughout the experiment. Overall, daily short-term temperature shocks to different developmental stages of parasitoids showed radical effects on emergence, fecundity, longevity, and sex ratio of the progeny. Therefore, releases of parasitoids should be conducted when they are in their pupal stages during the morning and evening in order to achieve their highest effectiveness for pest management.

## Introduction

*Trichogramma* (Hymenoptera: Trichogrammatidae) consists of over 600 species of egg parasitoids ( Park et al. 2000 ), many of which are used in biological control programs against a wide range of lepidopterous pests ( Corrigan and Laing 1994 ). *T. chilonis* Ishii is the most frequently encountered species in the Indian subcontinent and Southeast Asia (Nagarkatti and Nagaraja 1977), and *T. poliae* Nagaraja is a dominant parasitoid of the most destructive poplar defoliators, *Clostera cupreata* and *C. fulgurita* , in Himalayan regions of India ( Ahmad et al. 1999 , 2002). Both parasitoids are being deployed in mass in order to reduce lepidopteran pest populations in India.

Ecological compatibility of a biological control agent is an important aspect for successful agricultural pest control. The effectiveness of several biocontrol attempts has been reduced due to adverse climatic conditions. Temperature is a key factor affecting the abundance, distribution, and parasitic capabilities of egg parasitoids ( Bourchier and Smith 1996 ; Maceda et al. 2003). It has significant effects on developmental period and adult emergence of *Trichogramma* wasps ( Foerster and Foerster 2009 ). Short-term exposure to 35ºC temperature causes substantial mortality in *T. chilonis* (Nagarkati 1979). The functional response of the parasitoids is likely to change from one type to another with varying temperatures ( Kfir 1983 ; Wang and Ferro 1998 ; Mohaghegh et al. 2001), resulting in altered foraging behavior ( Zhang et al. 1983 ; Guo 1986 ). It has been reported that high temperature shocks on pupae and adults have significant impacts on fecundity, life duration, mobility, and efficacy of *Trichogramma* ( Chihrane et al. 1993; 1997; Chihrane and Lauge 1994 , 1996; Carriere and Boivin 1997 ).

Effects of temperature variation have been extensively studied on adult parasitoids, especially *Trichogramma* spp., but impacts on immature stages are often ignored. Additionally, several studies have been based on constant thermal exposure throughout the experimental period. However, subtropical countries like India, where the use of *Trichogramma* wasps as a biological control for agricultural pests is widely promoted, have variable temperatures during the day (usually six hours between 10:00 and 16:00). Regional temperature variability within the country ranges from less than 20ºC in hilly regions to over 40ºC in others. As a result of this variability, laboratory-reared parasitoids can be exposed to extreme temperature differences upon release, causing impacts that can affect their efficiency.

Simulation-based studies can give useful insight into the possible adverse effects of temperature variations on parasitoid performance. The present study was undertaken to evaluate the impacts of short-term exposure to extreme temperatures on the biological attributes of female *Trichogramma* and their immature progenies and to identify the best postembryonic stage for their field release.

## Materials and Methods

### Study location

The experiment was carried out in the Biological Control Laboratory of the Department of Entomology, G.B. Pant University of Agriculture and Technology, Pantnagar, Uttarakhand, India, from 2006 to 2009.

### Source of insect cultures

Both parasitoids ( *T. poliae* and *T. chilonis* ) were cultured and maintained at the Biological Control Laboratory since 2004 in controlled conditions (25–27ºC and 75 ± 5% RH) on the eggs of rice moth, *Corcyra cephalonica* Stainton (Lepidoptera: Pyralidae).

### Maintenance of insect culture for the experiment

*C. cephalonica* was reared on a standard diet containing maize, *Zea mays* L. (Poales: Poaceae). Fresh eggs of the factitious host were collected regularly and used for both culture and experiments. Parasitoids were considered to be in the egg stage immediately after parasitization of host eggs. On the third day of parasitization (i.e., before blackening of host eggs), the parasitoid was considered to be in the larval stage. From blackening of the host eggs until adult emergence, the parasitoid was considered to be in the prepupal and pupal stages ( Dahlan and Gordh 1996 ).

### Short-term exposure of immature stages of *Trichogramma* species to the variable temperatures

Paper strips containing 200 UV-light-treated eggs of *C. cephalonica* were exposed to large numbers of *T. poliae* and *T. chilonis* adults (about 500 females each) separately in glass test tubes (25 x 150 mm). Honey solution (10%) was provided as food to the adult parasitoids. After 24 hours of exposure, parasitized eggs were kept in an environmental chamber (OBROMAX, www.obromax.com ) at 20 ± 1ºC temperature and 75 ± 5% RH for the temperature shock period. After six hours of exposure, the parasitized eggs were kept at an ambient temperature (27 ± 1ºC) for of the rest of the day. This process was repeated daily until adult emergence. A similar protocol was followed subsequently for different temperature shocks (25 ± 1ºC, 30 ± 1ºC, 35 ± 1ºC, and 40 ± 1ºC). A constant photoperiod (16:8 L:D) was maintained throughout the experiment, and exposure to temperature shocks were always conducted under light conditions. Continuous rearing of parasitoids at an ambient temperature (27 ± 1ºC) was used as an untreated control.

The different temperature shocks were given as explained above on the third day of parasitization (larval stage) and on fifth day of parasitization (pupal stage). Individuals (immature stages only) exposed to thermal treatments formed the G _0_ generation, and the G _1_ generation was composed of their progeny.

Immediately after adult emergence, 20 mated females from each replication were isolated by treatment in small glass vials (12 x 75 mm). For studies on fecundity of females (G _0_ ), 100 UV-treated fresh eggs of host *C. cephalonica* were provided for parasitization at ambient conditions (27 ± 1ºC and 75 ± 5% RH) until death. Honey solution (10%) was provided as food to the adult female parasitoids. Fresh host eggs were provided daily, and the strips containing parasitized host eggs were separated. After five to six days of parasitization, fecundity was calculated. Observations of female longevity (G _0_ ) were recorded each morning. Females in the progeny (G _1_ ) were separated on the basis of antennae under stereoscopic microscope and sex ratio was calculated.

### Short-term exposure of egg-laying *Trichogramma* species to variable temperatures

Both parasitoids were reared under ambient conditions up to pupal stage. After the adult emergence, 20 newly emerged and mated females were isolated in separate glass vials and 100 host eggs (UV-treated) were provided daily. Honey solution (10%) was provided as food for adult females. These vials were kept in an environmental chamber at 20 ± 1º C temperature and 75 ± 5% RH. After six hours of exposure to temperature shock, the vials containing treated females were moved to ambient temperature (27 ± 1º C) for the rest of the day. Parasitoids were exposed to different temperature shocks each day for six hours until death. The females exposed to variable temperatures formed the F _0_ generation and the F _1_ generation was composed of their subsequent progenies. Daily fecundity (F _0_ ), longevity (F _0_ ) and sex ratio of their progeny (F _1_ ) were recorded using the above procedure. The same protocol was followed subsequently for different temperature shocks (25 ± 1ºC, 30 ± 1ºC, 35 ± 1ºC, and 40 ± 1ºC). Parasitoids reared at an ambient temperature (27 ± 1º C) without any temperature shocks were used as an untreated control.

### Experimental design and statistical analysis

The experiment on the effect of temperature shocks on immature stages of *Trichogramma* spp. was carried out with respect to two factors, temperature shock levels and life stage of parasitoids, with a completely randomized design. Data obtained in this study were analyzed using the general linear model (univariate analysis) in statistical software SPSS 14.0 (IBM, www.ibm.com ). Data obtained during the experiment regarding temperature effects on gravid *Trichogramma* females were analyzed using one way ANOVA ( *P* = 0.05). Tukey’s HSD test was applied to determine differences between means.

## Results

### Influence of variable temperature shocks on biological attributes of *T. poliae* and *T. chilonis*

**Figure 1.**
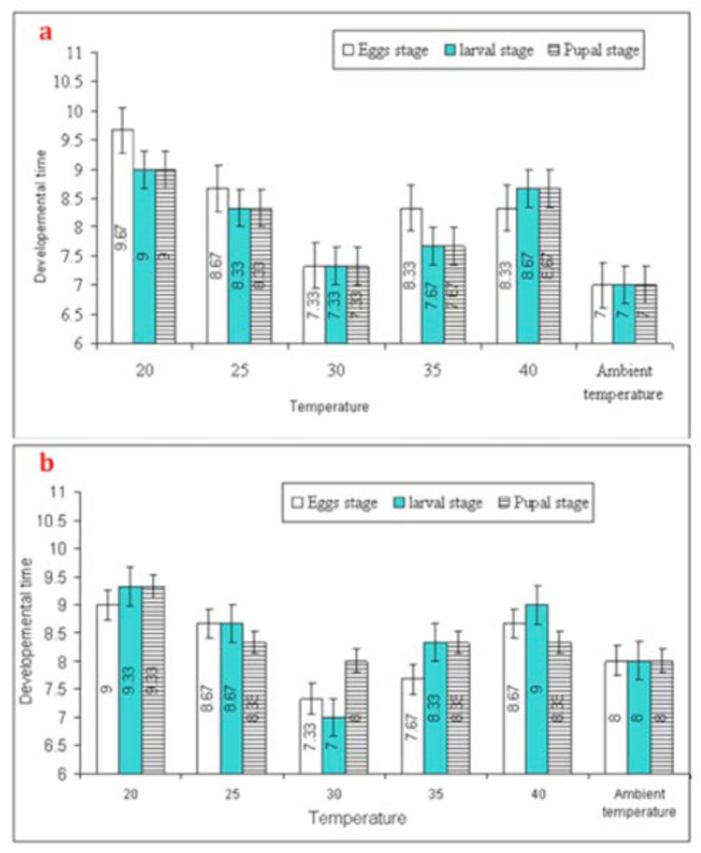
Mean (±SEM) development period (days) of *Trichogramma poliae* (a) and *T chilonis* (b) under the influence of various temperature shocks during immature stages. Bars having same arena with different letters indicate statistically significant differences ( *P* ≤ 0.05, Tukey’s HSD test). High quality figures are available online.

**Developmental period of parasitoids (G _0_ )** . Significant variation in developmental period of *Trichogramma* species was observed as a result of experimental temperature shocks ( Figure 1 ). Developmental period of *T. poliae* was remarkably extended (F = 9.15, *P* < 0.01, df = 5) due to both low and high temperature shocks. A similar trend was found in *T. chilonis* (F = 5.78, *P* < 0.01, df = 5). Differences in developmental period of *T. poliae* and *T. chilonis* were not significant when different stages of parasitoids were exposed to variable temperatures (F = 0.47, *P* = 0.62, df = 2, and F = 0.28, *P* = 0.75, df = 2, respectively).

**Figure 2.**
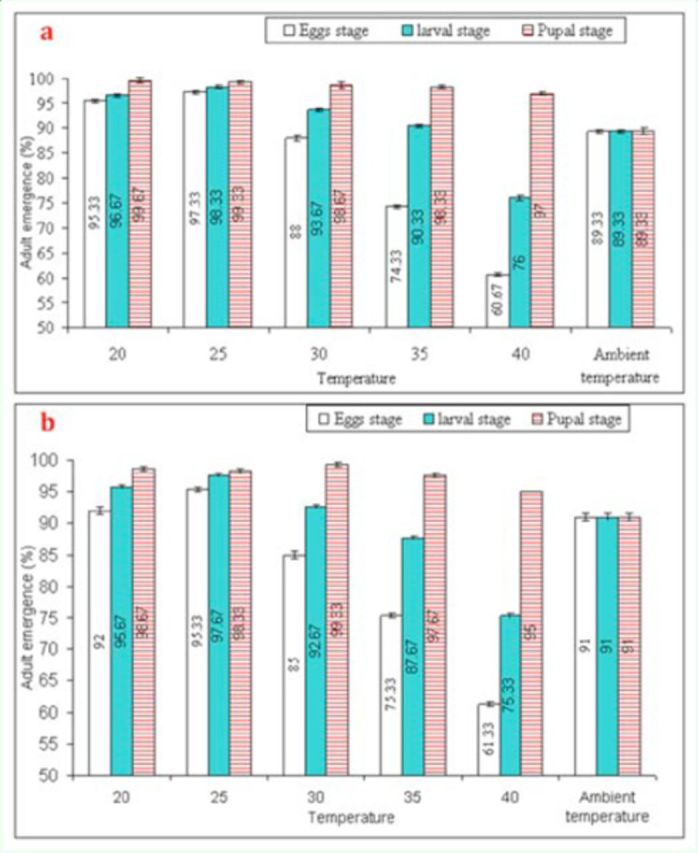
Mean (±SEM) adult emergence (%) of *Trichogramma poliae* (a) and *T. chilonis* (b.) under the influence of various temperature shocks during immature stages. Bars having same arena with different letters indicate statistically significant differences ( *P* ≤ 0.05, Tukey’s HSD test). High quality figures are available online.

**Adult emergence of parasitoids (G _0_ ).** Adult emergence of *T. poliae* and *T. chilonis* was also significantly affected (F = 1148.24, *P* < 0.01, df = 5, and F = 934.58, *P* < 0.01, df = 5, respectively) as a result of daily short-term exposure to higher temperatures ( Figure 2 ). Furthermore, adult emergence was significantly affected when egg-stage *T. poliae* (F = 1682.17, *P* < 0.01, df = 2) and *T. chilonis* (F = 1600, *P* < 0.01, df = 2) were exposed. Significant differences were also noticed between different temperature shocks and life stages of *T. poliae* (F = 360.77, *P* < 0.01, df = 10) and *T. chilonis* (F = 251.51, *P* < 0.01, df = 10).

**Figure 3.**
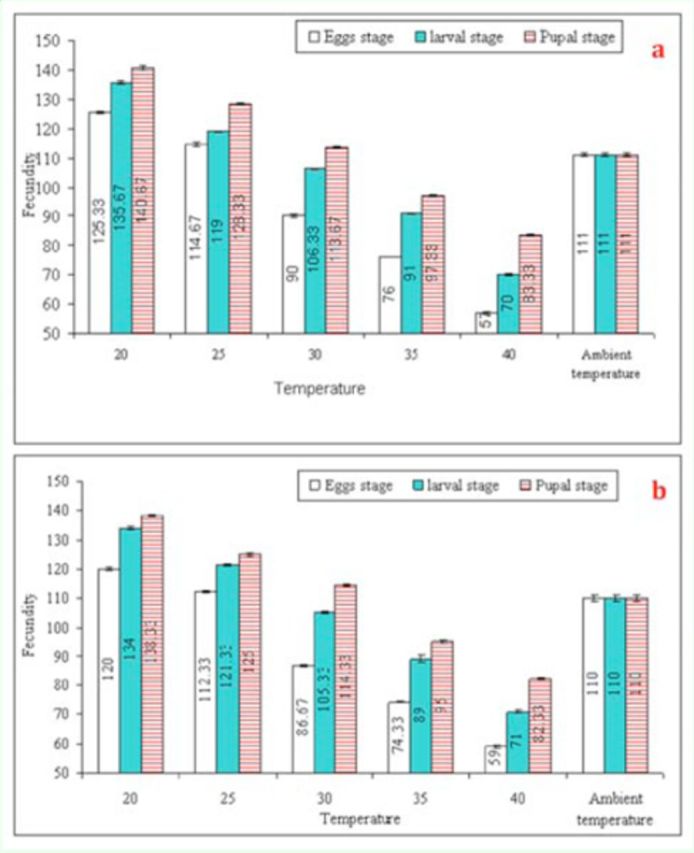
Mean (±SEM) lifetime fecundity (no. of host parasitized per female) of *Trichogramma poliae* (a) and *T. chilonis* (b) under the influence of various temperature shocks during immature stages. Bars having same arena with different letters indicate statistically significant differences *(P ≤* 0.05, Tukey’s HSD test). High quality figures are available online.

**Figure 4.**
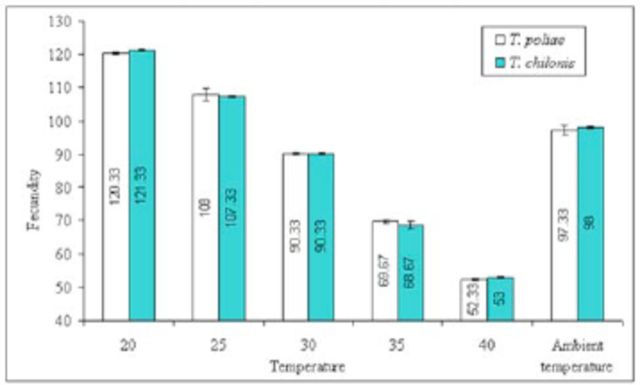
Mean (±SEM) lifetime fecundity (no. of host parasitized per female) of two *Trichogramma* species at various temperature shocks to the egg-laying females. Bars having same arena with different letters indicate statistically significant differences ( *P* ≤ 0.05, Tukey’s HSD test). High quality figures are available online.

Fecundity of parasitoids (G _0_ and F _0_ ). Fecundity of *T. poliae* (G _0_ ) and *T. chilonis* (G _0_ ) was significantly reduced (F = 2294, *P* < 0.01, df = 5 and F = 3176.98, *P* < 0.01, df = 5, respectively) as a result of high temperature shocks ( Figure 3 ). The effect was more severe when the *T. poliae* (F = 618.49, *P* < 0.01, df = 2) and *T. chilonis* egg-stage (F = 996.61, *P* < 0.01, df = 2) was exposed than when the larval and pupal stages were exposed. A significant reduction in fecundity was also observed when egg-laying females (F _0_ ) of *T. poliae* (F = 675.98, *P* < 0.01, df = 5) and *T. chilonis* (F = 796.22, *P* < 0.01, df = 5) were exposed to various temperature shocks ( Figure 4 ).

### Female longevity of parasitoids (G _0_ and F _0_ ).

**Figure 5.**
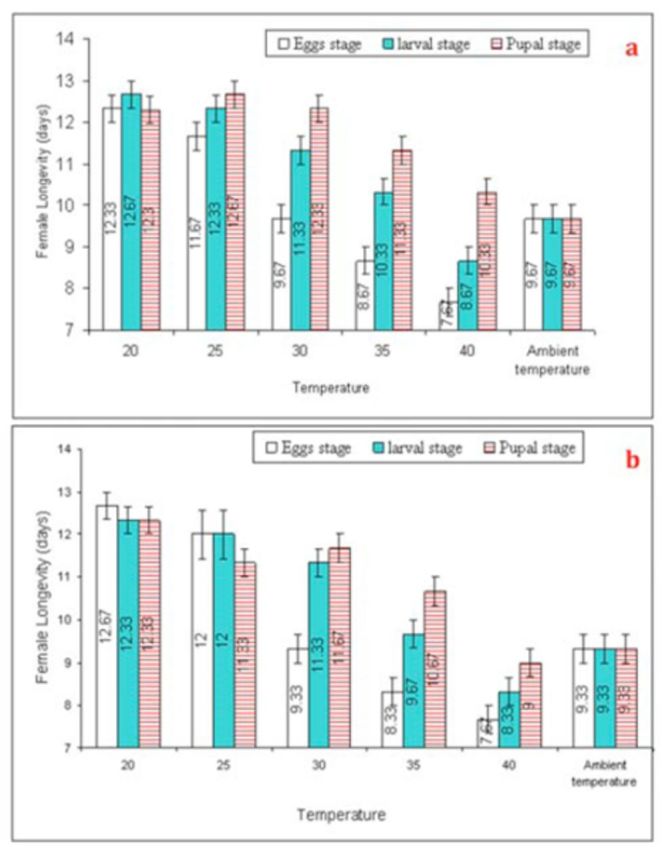
Mean (±SEM) longevity (days) of *Trichogramma poliae* (a) and *T. chilonis* (b) under the influence of various temperature shocks during immature stages. Bars having same arena with different letters indicate statistically significant differences ( *P* ≤ 0.05, Tukey’s HSD test). High quality figures are available online.

**Figure 6.**
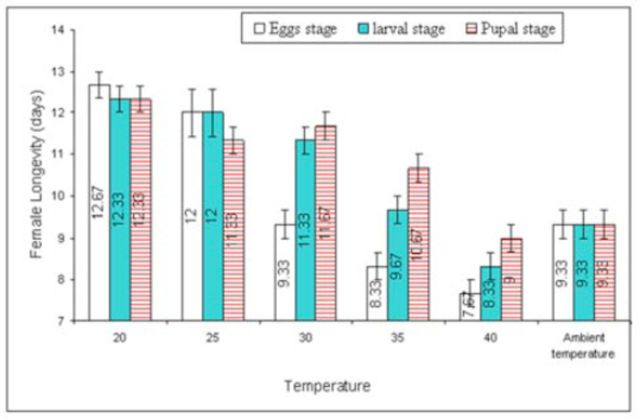
Mean (±SEM) longevity (days) of two *Trichogramma* species at various temperature shocks to egg-laying females. Bars having same arena with different letters indicate statistically significant differences ( *P* ≤ 0.05, Tukey’s HSD test). High quality figures are available online.

The life spans of female *T. poliae* (G _0_ ) and *T. chilonis* (G _0_ ) were considerably reduced (F = 53.73, *P* < 0.01, df = 5. and F = 49.97, *P* < 0.01, df = 5, respectively) from short-term exposure to 40º C temperature shocks ( Figure 5 ). Longevity of female *T. poliae* (G _0_ ) and *T. chilonis* (G _0_ ) was higher when exposed to higher temperatures at the pupal stage (F = 11.40, *P* < 0.01, df = 2, and F = 7.54, *P* < 0.01, df = 2, respectively) than the larval and egg stage. Increased intensity and frequency of thermal shocks caused significant effects on longevity of *T. poliae* (F _0_ ) (F = 16.89, *P* < 0.01, df = 5) and *T. chilonis* (F _0_ ) (F = 53.40, *P* < 0.01, df = 5) ( Figure 6 ).

**Figure 7.**
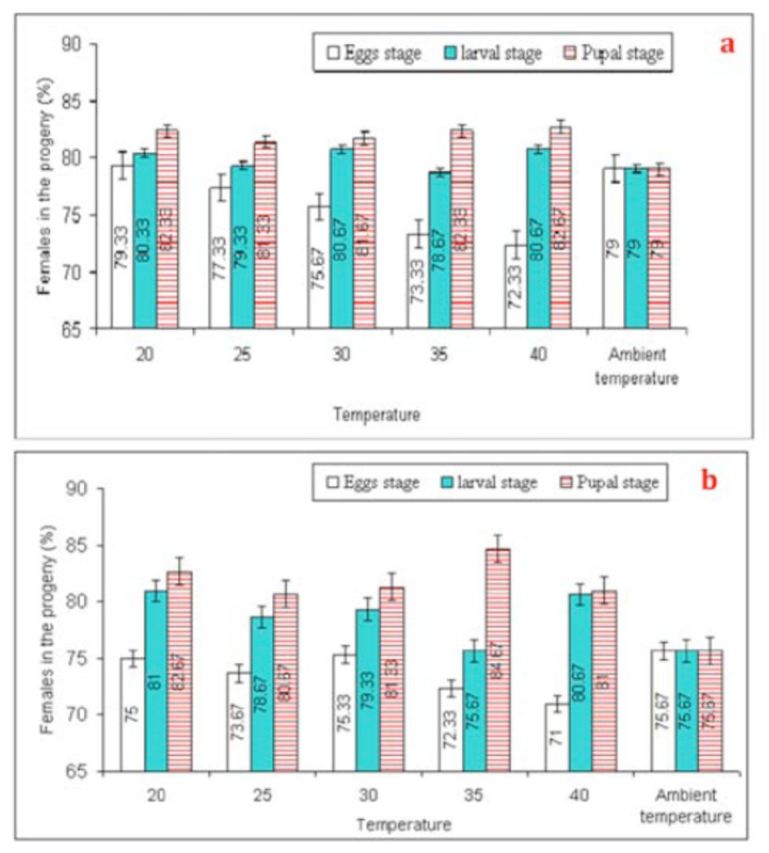
Mean (±SEM) values of % females in the progeny of *Trichogramma poliae* (a) and *T. chilonis* (b) under the influence of various temperature shocks during immature stages. Bars having same arena with different letters indicate statistically significant differences ( *P* ≤ 0.05, Tukey’s HSD test). High quality figures are available online.

**Figure 8.**
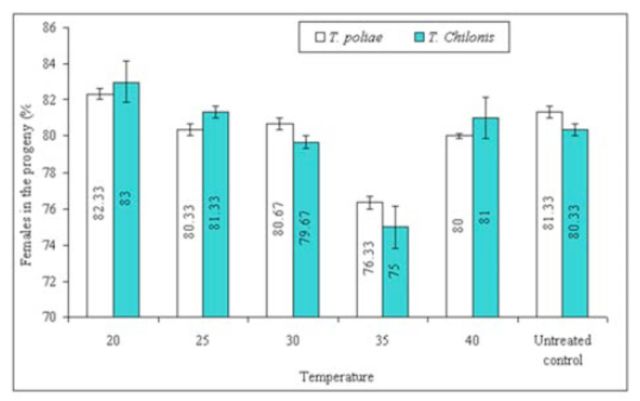
Mean (±SEM) values of % females in the progeny of two *Trichogramma* species at various temperature shocks to egg-laying females. Bars having same arena with different letters indicate statistically significant differences ( *P* ≤ 0.05, Tukey’s HSD test). High quality figures are available online.

**Sex ratio of the progeny (G _1_ and F _1_ ).** No significant differences were noticed in sex ratio of the progeny (G _1_ ) of *T. poliae* (F = 0.50, *P* = 0.77, df = 5) or *T. chilonis* (F = 0.73, *P* = 0.60, df = 5); when their mothers (G _0_ ) were exposed to various temperature shocks ( Figure 7 ). However, the sex ratio of the progeny of *T. poliae* and *T. chilonis* (G _1_ ) decreased considerably (F = 9.93, *P* < 0.01, df = 2, and F = 11.38, *p* < 0.01, df = 2, respectively) under high temperature shocks at the egg stage, fa-voring females ( Figure 7 ). Furthermore, the sex ratio of *T. poliae* (F _1_ ) was significantly affected (F = 13.37, *P* < 0.01, df = 5) as a result of various temperature shocks to egg-laying females (F _0_ ) ( Figure 8 ). A parallel trend was observed in *T. chilonis* (F _1_ ) (F = 10.24, *P* < 0.01, df = 5) ( Figure 8 ). Interestingly, sex ratio of both the parasitoid species was female-biased under all temperature shock levels.

### Discussion

Temperature is the most important variable impacting the development of invertebrates. The developmental period of insects largely depends on environmental temperatures. In this study, both high and low temperature shocks extended the developmental period of *Trichogramma* species. Changes in enzyme levels, the properties of the cell membrane, as well as other physiochemical changes induced by fluctuating temperatures may have extended the developmental period of insects ( Wang et al. 2004 ). The relationship between temperature and development is often linear throughout the range of temperatures suitable for insect development and becomes sigmoid throughout the temperature ranges unsuitable for insect development ( Andrewartha and Birch 1954 ; Lactin et al. 1995 ; Arbab et al., 2006 ). Therefore, the results of our study are in agreement with several reports on other *Trichogramma* species ( Butler and Lopez 1980;Calvin et al. 1984;Naranjo 1993 ; Park et al. 2000 ; Suverkropp et al. 2001 ). Extended developmental period under fluctuating temperatures may also be attributed to reduced metabolic conditions for prolonged periods (Hassan et al. 1990; Tezze and Botto 2004 ).

Developmental period was least affected when parasitoids were exposed in the pupal stage. This stage has been suggested to be more tolerant of temperature extremes. Interestingly, the developmental period of *T. poliae* was relatively less affected, even under higher temperatures, possibly a result of originating from a warmer climate ( Nagaraja 1973 ).

When temperature deviates from its optimum, mortality rate increases and premature mortality is observed among individuals ( Chihrane and Lauge 1996 ). This inverse relationship of temperature with emergence of *Trichogramma* has been supported by several studies ( Ram and Sharma 1977 ; Lopez and Morrison 1980 ; Chihrane et al. 1993 ; Miura and Kobayashi 1993 ; Park et al. 2000 ). Reduced *Trichogramma* emergence has also been reported even at 32ºC ( Consoli and Parra 1995 ), 35ºC ( Harrison et al. 1985 ; Scholler and Hassan 2001 ; Ayvaz et al. 2008 ), and 42ºC ( Fye and Larsen 1969 ). Lower emergence at higher temperatures might be due to higher mortality of parasitoids inside the host egg itself. Under such conditions, parasitoids may be unable to perforate the chorion of host egg, ultimately blocking emergence ( Chihrane and Lauge 1996 ).

In this study, adult emergence was more significantly affected when parasitoids were exposed in the egg-stage, as opposed to beginning exposure at the larval and pupal stages. Desiccation of host eggs, prolonged heat stress, and sudden changes in metabolic activities might have affected parasitoid development, which ultimately reduced adult emergence. Insect pupae are usually comparatively hardier than eggs and larvae; therefore, temperature-induced effects on the parasitoid pupae were less significant, even up to 40 ± 1ºC. Pupal stages in this study were given fewer thermal shocks than the other life stages. However, extreme temperature exposure of pupal-stage *T. pretiosum* ( Lopez and Morrison 1980 ) and *T. brassicae* ( Chihrane and Lauge 1996 ) significantly reduced the emergence of adults (Hassan et al. 1988; Steidle et al. 2001 ; Mansfield and Mills 2002 ). This may be due to their originating from a colder climate. The parasitoids used in this study originated from a tropical climate.

Short-term exposure to high temperatures also had significant impacts on field parasitism of crop pests by *T. ostriniae* ( Wang et al. 1997 ). A negative correlation between temperature and fecundity has been repeatedly reported in many parasitoids ( Sorokina 1978 ; Zaslavskiy and May Phu Qui 1982 ; Atamirzaeva 1991;Pavlik 1992 ; Scott et al. 1997 ; Wang and Ferro 1998 ). In our findings, fecundity of parasitoids was considerably reduced due to high temperature shocks, in congruence with earlier reports on *Trichogramma* species (Calvin et al. 1984; Chihrane et al. 1993 ; Chihrane and Lauge 1996 ; Reznik et al. 1997 ; Maisonhaute et al. 1999; Singh and Ram 2006 ; Reznik and Vaghina 2006a, b ; Ayvaz et al. 2008 ). Reduced fecundity is linked to the disruption of ovarian function as a result of high temperatures during postembryonic development. Possibilities of some pathological effects have also been reported (partial male sterility and female oogenesis suppression) in *T. brassicae* due to heat shocks and their ultimate influence on oviposition ( Chihrane et al. 1993 ; Chihrane and Laugé 1994 , 1996). In this study, the pupal stage was found to be relatively tolerant, which could be due to their hardiness and fewer thermal shocks, as previously described.

Longevity was also significantly reduced due to high temperature shocks given during immature and adult stages. However, life span was comparatively longer when parasitoids were exposed in their pupal stages. Shortened longevity could be due to shrinkage or desiccation of host eggs, which ultimately result in the formation of weak parasitoids. Usually, malnourished parasitoids (food-deprived females) live shorter life spans than well-fed parasitoids ( Reznik et al. 1997, 2003 ). In this investigation, longevity was inversely related to temperature shocks, suggesting that temperature plays a significant role on overall parasitization. Therefore, the results are in accordance with several reports on other *T.* species ( Lund 1938 ; Ram and Sharma 1977 ; Pak and Oatman 1982; Harrison et al. 1985 ; Rogor 1988; Chihrane et al. 1991 ; Bourchier and Smith 1996 ; Babi and Nabham 1998 ; Ayvaz et al. 2008 ).

Thermal fluctuations given during immature stages (G _0_ ) had lesser effects on the sex ratio of the progeny (G _1_ ) and less effects on the pupal stage compared to egg and larval stages. Tolerance of *T. chilonis* pupae to extreme temperatures has been reported ( Singh and Ram 2006 ), whereas sex ratio was relatively unchanged due to temperature variations. However, the sex ratio of the progeny of both parasitoids (F _1_ ) was significantly affected due to thermal shocks in this study. The effects on the sex ratio of the progeny were determined by the rearing temperature during the mother’s development (Bower and Stern 1996). In our study, the mothers (F _0_ ) were reared at ambient conditions and exposed to variable temperatures during the adult stage only. This might have affected the sex ratio of the progeny (F _1_ ). A similar relationship between temperature and sex ratio has been observed in several *Trichogramma* species (Parker and Pinnell 1971; Harrison et al. 1985 ; Consoli and Parra 1995 ). In our experiment, a female-biased sex ratio consistently resulted when the mother was exposed to different temperature shocks, a trend that has been as reported in many trichogrammatids ( Harrison et al. 1985 ; Haile et al. 2002 ; Saljoqi and YuRong 2004 ; Foerster and Foerster 2009 ).

### Conclusion

The results of our study suggest that high and low temperature shocks have considerable impacts on female parasitoid longevity, fecundity, adult emergence, and sex ratio of their progeny. Although the parasitoids in this study were found to survive and reproduce at temperatures up to 40ºC, their releases for pest control would be more effective between 23 ± 1ºC and 26 ± 1ºC. Parasitoids should be released just before their emergence (during the pupal stage) and preferably during the morning or evening in order to achieve the highest level of effectiveness for better pest management.
